# Challenges in Gynecological Cancer: Biology, Diagnosis, Surgical, and Medical Treatment

**DOI:** 10.1155/2015/787080

**Published:** 2015-06-22

**Authors:** Ignacio Zapardiel, Shalini Rajaram, Elisa Piovano, Marco Petrillo

**Affiliations:** ^1^Gynecologic Oncology Unit, La Paz University Hospital, IdiPAZ, 28046 Madrid, Spain; ^2^Department of Obstetrics and Gynecology, University College of Medical Sciences and Guru Teg Bahadur Hospital, Delhi 110092, India; ^3^Department of Surgical Sciences, University of Turin and Obstetrics & Gynecology Unit, Martini Hospital, 10141 Turin, Italy; ^4^Gynecologic Oncology Unit, Policlinico Universitario Agostino Gemelli, 00168 Rome, Italy

The last few years have witnessed a better understanding of molecular events that cause gynecological cancers and new insights are changing the practice of gynecologic oncology. The implementation of new concepts into daily clinical practice may improve treatment received by patients, and, more importantly, it could influence their survival and quality of life, which should be our final endpoint. The aim of this special issue was to synthetize several advances in gynecological cancer, both molecular aspects and their clinical application. We have included six research papers, half of which focused on basic research aspects and the other half focused on clinical management and usefulness.

U. Andergassen et al. report in 235 breast cancer samples that glycosylation patterns identified by immunohistochemistry could be used as a marker of early tumorigenesis since glycosyltransferases are related to smaller breast cancer tumors as well as well-differentiated ones. The Chinese group of X. Zhao et al. studied levonorgestrel effects on endometrial cancer, which is a topical subject, considering the increasing social demand for fertility preservation management for gynecological cancers. They found a time-dependent inhibition of cell proliferation with an increase of apoptosis in both human endometrial stromal cells and glandular cells by means of the upregulation of Cx43 expression in the gap junctional intercellular communication. Another basic research article focuses on the study of microvessel density and its survival implications among women with uterine leiomyosarcoma. M. Bobiński et al. did not find among their 50 patients studied a significant correlation between the microvessels density and patients' overall and 2-year survival. Their conclusions suggest that additional mechanisms apart from angiogenesis should be considered and studied in future research on uterine leiomyosarcoma.

A. Wong and J. Ngeow reviewed the influence of pathological features in the risk assessment of hereditary endometrial cancer syndromes. Specifically they studied the microscopic features as well as immunohistochemical and polymerase chain reaction based tests for DNA mismatch repair and PTEN gene mutations to establish a more targeted and cost-effective approach to those patients with hereditary syndromes. With the aim of solving the unanswered question of the early diagnosis of ovarian cancer, A. Barua et al. examined the feasibility of using interleukin-16 targeted ultrasound imaging for the detection of ovarian tumors. They found significant enhanced ultrasound signal intensity among ovarian cancers compared to other ovarian pathologies, both in early and in late stages of the disease. These findings could improve the early diagnosis of ovarian cancer by ultrasonographic scan. Another article includes novel information on the clinical usefulness of immunochemistry in the differential diagnosis of an uncommon disease, specifically the complete mole. S. Sasaki et al. studied the staining of p57^kip2^ in 14 cases of gestational trophoblastic disease. They demonstrated its usefulness for the differential diagnosis between complete and partial mole which is much cheaper than the current gold standard DNA analysis.

To conclude, we consider the current special issue a good opportunity to update our knowledge on the latest research of two very common cancers such as breast and endometrial cancer. Interesting articles on uterine sarcoma, ovarian cancer, and gestational trophoblastic disease may aid the practicing gynecologic oncology in decision making.

*Ignacio Zapardiel* *Shalini Rajaram* *Elisa Piovano* *Marco Petrillo*

